# Effect of Fermented Rapeseed Meal on the Gastrointestinal Microbiota and Immune Status of Rabbit (*Oryctolagus cuniculus*)

**DOI:** 10.3390/ani11030716

**Published:** 2021-03-06

**Authors:** Łukasz Wlazło, Dorota Kowalska, Paweł Bielański, Anna Chmielowiec-Korzeniowska, Mateusz Ossowski, Marcin Łukaszewicz, Anna Czech, Bożena Nowakowicz-Dębek

**Affiliations:** 1 Department of Animal Hygiene and Environmental Hazards, University of Life Sciences in Lublin, Akademicka 13, 20-950 Lublin, Poland; lukasz.wlazlo@up.lublin.pl (Ł.W.); anna.korzeniowska@up.lublin.pl (A.C.-K.); bozena.nowakowicz@up.lublin.pl (B.N.-D.); 2 National Research Institute of Animal Production, Krakowska 1, 32-083 Balice, Poland; pawel.bielanski@izoo.krakow.pl; 3 Department of Biotransformation, Faculty of Biotechnology, University of Wroclaw, F. Joliot-Curie 14A, 50-383 Wrocław, Poland; marcin.lukaszewicz@uwr.edu.pl; 4 Department of Biochemistry and Toxicology, University of Life Sciences in Lublin, Akademicka 13, 20-950 Lublin, Poland; anna.czech@up.lublin.pl

**Keywords:** *Oryctolagus cuniculus*, rabbit, fermented rapeseed meal, GIT microbiota, immunoglobulin

## Abstract

**Simple Summary:**

The unique digestive properties of rabbits consist of highly specialised communities of intestinal microbes that, unfortunately, make them susceptible to metabolic diseases. This is why breeders, to improve the functions of the digestive tract, often use special feed additives, i.e., probiotics, prebiotics or synbiotics. The need to become independent from soybean meal (SBM), which is currently the basic source of protein in animal nutrition, and the need to stimulate the gastrointestinal tract (GIT), has increased interest in fermented components that have a positive effect on the intestinal microbiota and are a source of valuable protein. In this study, the impact of the diversified proportion of fermented rapeseed meal (FRSM) in the diet of rabbits on the immune parameters and the microbiota of the digestive tract was assessed. The reducing effect of the tested feed component against coliform bacteria and *Escherichia coli* within the small intestine and colon of animals and the anaerobic biota of *Clostridium perfringens* in the duodenum and cecum of animals was observed while in the duodenum—an increase in the beneficial biota of lactic acid bacteria. The conducted analysis also showed many complex correlations between the number of intestinal microbiota groups and the level of immunoglobulins. The results of the conducted research indicate that FRSM, in addition to valuable nutritional values, may play an important probiotic role in the GIT of rabbits. Research of this type is especially important in terms of reducing the use of antibiotics for therapeutic purposes through nutritional prevention of animals.

**Abstract:**

The present study was conducted to determine the effect of the use of varying amounts of fermented rapeseed meal in diets for rabbits on the immune status and microbiota of segments of the GIT. Forty 35 day old rabbits used in the experiment were assigned to four groups: the control group (group C) were fed a standard diet and the experimental received 4%, 8% or 12% fermented rapeseed meal (included in place of standard soybean meal). Class A, G and M immunoglobulins were determined in the blood plasma. In the food content collected after slaughter, microbiological parameters were determined for individual sections of the digestive tract. Rabbits from the groups receiving a diet with an increased proportion of fermented rapeseed meal (8% or 12%) had lower concentrations of anaerobic bacteria and *Escherichia coli* in the intestinal contents. Research has shown that the increase in intake of fermented rapeseed meal was correlated with an increase in the correlations between the immunoglobulin level and the size of the microbial population in the GIT. In light of the presented results fermented rapeseed meal, by supplying valuable bioactive substances, appears to be a good component in the diet of rabbits, enhancing immune system development and helping to prevent disturbances of the gut microbiota.

## 1. Introduction

The complex microbiota of the gastrointestinal tract (GIT) of animals plays an important role in the digestion of nutrients and protection against infections resulting from the presence of pathogens and environmental bacteria. It also functions as a barrier against harmful exogenous substances and ensures normal metabolic, immune and neurological functions in the host. The GIT is involved in numerous physiological processes, from nutrition to behavioural and stress responses [1]. The unique digestive properties of rabbits and specialised microbial communities can help them to adapt to fibre-rich foods but often make them susceptible to metabolic diseases. For this reason, control and modulation of the microbiota of rabbits is an important aspect of breeding practice. Digestive problems can be controlled through the immunostimulatory activity of the microbiota and competitive exclusion, which is particularly important in young animals after weaning.

As in the case of all mammals, the introduction of new species to the GIT of rabbits is determined by ecological succession. Microorganisms, competing for nutrients, colonise an ecological niche and consume all food resources. These bacteria are able to inhibit the growth of competing bacteria by producing antimicrobial substances [2]. A community of intestinal microbes is formed by random colonisation from the surrounding meta-community (the mother, bed, cage, air, etc.) and is highly variable between individuals up to the age of 49 days [3]. By the age of 70 days, the composition of the caecal microbiota is highly homogeneous and shows a certain degree of stabilisation. In rabbits, a disturbance of this normal microbiota, known as dysbiosis, is widely considered to be the cause of enteritis with symptoms of diarrhoea, followed by dehydration and potentially death. The aetiopathogenesis of intestinal inflammation in rabbits is complex, and there are generally multiple factors. Enteric pathogens, such as *Escherichia coli*, *Clostridium spiroforme*, *Lawsonia intracellularis*, *Clostridium piliforme*, *Salmonella* spp., rotaviruses, coronaviruses, parvoviruses and astroviruses, are most often found in individuals with diarrhoea. However, there is still little information on the factors that initiate it, as infection with these microbes is not synonymous with disease. Most cases of enteritis in rabbits are caused by a combination of multiple environmental factors and infectious agents, including a low-fibre diet, an overall weakened state of health, stress associated with management and inadequate welfare, and age, as well as the presence of one or more potentially pathogenic microbes [2,4].

During microbiological fermentation in favourable intestinal conditions, rabbits receive products that stimulate colonization by symbiotic microbes. In rabbits, this is referred to as a combined model of competition and cooperation of the gut microbiota. However, the balance of this ecosystem is fragile and can be destroyed during digestive disorders [5]. An appropriate diet therefore plays a key role in prevention, which is why breeders often use special feed additives, such as probiotics, prebiotics or synbiotics, to improve gastrointestinal function. These additives, due to the fact of their antagonistic effect on pathogenic and opportunistic microbes, are the subject of growing interest among livestock breeders.

Improvement in the efficiency of digestion through optimisation of the composition of the microbiota directly improves nutrient digestibility and stimulates immune processes, increasing the profitability of production. This is one of the main reasons that breeders are searching for alternative solutions involving administration of probiotic microbes with feed in the form of monocultures or a mixture of different strains. The most commonly used probiotics in breeding practice include species of the genera *Lactobacillus*, *Enterococcus*, *Pediococcus*, *Bifidobacterium*, *Saccharomyces* and *Bacillus*. Work is also being conducted on recombined probiotics, which are among the most innovative biomedical applications of genetically modified organisms [6].

Rabbits, like other farmed animals, are fed complete pelleted feed containing soybean meal (SBM) as the main source of protein. In some parts of the world, especially where soybean is not cultivated, recent years have seen a trend towards elimination of SBM from feed for most livestock animals. This is due to the desire to become independent of imported feed or to concerns about products containing genetically modified organisms [7]. In the diet of rabbits, the possibility of replacing SBM with other high-protein plant-based feeds has been studied. The use of dried kernels of barley, wheat and maize, white lupin seeds [8,9], peas [10,11,12] or other plants of the Fabaceae family [13,14] has been shown to have a beneficial effect on production efficiency. However, a large share of these ingredients in the diet results in health problems associated with the presence of anti-nutrients (e.g., tannins, antitrypsin factor, haemagglutinin, α-galactosides and alkaloids) or problems involving the balancing of the diet [15,16,17].

Fermented protein components are currently a subject of great interest. Owing to their synergistic effect involving stabilisation of the gut microbiota and their valuable nutritional properties, they are becoming a sought-after bioproduct in the feed market. At the same time, efforts to limit the use of animal feed containing genetically modified organisms (GMOs) have prompted the search for an alternative source of easily digestible protein. A new material that meets these expectations may be fermented rapeseed meal (FRSM). Due to the microbial fermentation process, rapeseed meal (RSM), on the one hand, loses its anti-nutritional substances and, on the other hand, acquires probiotic properties. Moreover, it becomes a source of sulphur-containing amino acids, more easily digestible protein, digestive enzymes, and antioxidant compounds. In research in pigs, its inclusion in the diet has been shown to improve nutrient digestibility, resulting in improved growth performance. At the same time, by reducing unfavourable gut microbes and stimulating immune processes, FRSM has a prophylactic function and positively affects animal health [18].

Bacteria of the genus *Bacillus*, used in fermentation of plants, can perform a probiotic function, and the products of their metabolism can beneficially modulate immune system activity in animals [18,19]. De-Yu Hung et al. [19] demonstrated that *Bacillus* bacteria have a positive effect by alleviating diarrhoea and reducing the number of gut pathogens. The studies cited show that FRSM can partially replace SBM in diets for monogastric animals and can be a valuable additive stimulating immune processes in the body and, thus, improve the condition of animals.

The available literature lacks studies on the possibility of using FRSM in the diet of rabbits. Moreover, there are few studies characterising the gut microbiota of rabbits, and these often focus only on individual segments of the GIT. Therefore, the present study was conducted to determine the effect of the use of varied amounts of FRSM in diets for rabbits on the immune status and microbiota of segments of the GIT, i.e., the duodenum, small intestine, caecum and colon.

## 2. Materials and Methods

### 2.1. Preparation of FRSM

RSM was fermented using the strain Bacillus subtilis 87Y from the strain collection of InventionBio Ltd. (Bydgoszcz, Poland). The bacteria were multiplied on Lysogeny Broth (LB) medium (10 g/L NaCl, 10 g/L peptone, 5 g/L yeast extract), from which a bacterial suspension of OD 600 = 0.1 was obtained in MIM1 medium (8.4 g/L Na_2_HPO_4_, 3.9 g/L NaH_2_PO_4_, 2.3 g/L urea, 0.5 g/L MgSO_4_, 60 g/L saccharose, 1.2 mg/L FeSO_4_, 1.6 mg/L CuSO_4_, 5 mg/L MnSO_4_), [20]. Prior to fermentation the RSM was sterilised for 15 min and then inoculated with the previously prepared bacterial suspension in a 1:1 ratio while maintaining 50% moisture by adding sterile water and aerating at 50 L/min. Fermentation was carried out for 24 h at 37 °C with continuous mixing (20 rpm), after which the RSM was dried to 10–11% moisture with a fluid bed dryer.

### 2.2. Experimental Animals and Diet

Forty 35 day old rabbits (*Oryctolagus cuniculus*) were used in the experiment. The rabbits were crosses of two breeds: New Zealand White, a typical meat breed, and Popielno White.

The animals were assigned to four groups of 10 each, with similar body weights in each group. Each group comprised 5 females and 5 males. The animals were kept in cages of two, which gave five repetitions within each group. The animals in the control group (group C) were fed a standard diet. The experimental groups received 4% (group FR4), 8% (group FR8) or 12% (group FR12) FRSM, (Table 1). FRSM was included in place of standard SBM, and the chemical composition of the feed ration in all groups was balanced according to standards for feeding fur-bearing animals (Table 1), [21]. During the experiment, the animals were under the constant supervision of the farm veterinarian, were fed ad libitum and had free access to drinking water.

In each group, 5 animals of each sex were kept in two-storey wire mesh cages equipped with a feeder and a nipple drinker, in a closed, climate-controlled building. The rabbits received feed containing FRSM from weaning at 35 days of age until slaughter at 120 days. All tests on animals were performed with the approval of the Local Ethics Committee for experiments on animals (approval no. 65/2020).

### 2.3. Experimental Procedures and Sample Collection

#### 2.3.1. Chemical Analysis of FRSM and Feed Mixtures

The chemical composition of the FRSM and of the diets for animals in groups C, FR4, FR8 and FR12 (i.e., the content of total protein, dry matter and crude ash) was analysed according to Latimer [23]. We also determined the quantitative composition of amino acids (Lys, Met and Cys) by ion-exchange chromatography with spectrophotometric detection (IEC-Vis). The content of Ca^2+^ and Na^+^ was determined by atomic absorption spectrometry [23]. Total phosphorus content was determined by spectrometry according to Fiske and Subbarow [24].

In addition, also determined in the diet and fermented components were the content of phytate phosphorus according to Oberleas [25], lactic acid content according to Taylor [26], glucosinolates according to ISO 10633-1 [27] and tannins according to Canbaş et al. [28].

The analyses of FRSM, RSM and feeds were carried out in three batches and in duplicate.

#### 2.3.2. Experimental Procedures and Sample Collection

##### Collection of Biological Material for Analysis

At the end of the experiment, when the animals were 120 days old, 6 rabbits (3 male and 3 female) with average body weight/group were selected for slaughter.

Before slaughter, at 119 days of age, 6 rabbits (3 male and 3 female) with average body weight/group were selected for blood collection. Blood was drawn following local anaesthesia from the marginal ear vein into Monovette tubes with a clot activator (Sarstedt). The blood was centrifuged in a laboratory to obtain plasma for determination of immunoglobulins.

At the end of the experiment, at 120 days of age, the animals from which blood had been taken were slaughtered. The animals were decapitated after being stunned by a blow to the head, owing to which the body of the animal was relaxed and sensitivity to pain was completely and immediately eliminated. The animals were slaughtered in accordance with Council Regulation (EC) no. 1099/2009 of 24 September 2009 on the protection of animals at the time of killing [29].

Immediately after an incision was made in the abdominal wall, the entire GIT tract was removed from each carcass, and then the intestinal contents from each segment of the GIT were sampled in sterile conditions using an MSC Advantage class II laminar safety cabinet with HEPA filters (Thermo Scientific, Waltham, MA, USA). From each part of the GIT about 10 g of intestinal contents were sampled. The samples were homogenised with a CAT Unidrive X1000 handheld homogeniser (Deerfield, IL, USA) for 60 s at a blade speed of 4000 rpm.

##### Blood Analysis

Class A, G and M immunoglobulins were determined in the blood plasma using ELISA assays (Genorise Scientific Inc., Glen Mills, PA, USA).

##### Microbiological Analysis

From the homogenised intestinal contents, 20 g of material was weighed out and placed in sterile bottles containing 180 mL of Ringer solution. The solution was shaken for 5 min and left to settle for 15 min. Then ten-fold serial dilutions were prepared in Ringer solution and plated on previously prepared Petri dishes with an appropriate microbiological medium. The following were determined in the material:

1. The total number of mesophilic aerobic bacteria, on enriched agar medium for 48 h at 37 °C (BTL Ltd., Łódź, Poland);
2. The total number of fungi, on Sabouraud agar, for 5–7 d at 25 °C (BTL Ltd., Łódź, Poland);
3. The total number of coliform bacteria on Endo LES agar, for 24 h at 37 °C (BTL Ltd., Łódź, Poland);
4. The total number of *Escherichia coli*, on mFC agar for 18–24 h at 44 °C (BTL Ltd., Łódź, Poland);
5. The total number of *Clostridium perfringens*—sulphate-reducing bacteria growing in anaerobic conditions, on iron sulphite agar (TSC) for 48 h at 37 °C (BioMerieux, Marcy l’Etoile, France);
6. The total number of lactic acid bacteria of the genus *Lactobacillus*, on MRS agar for 3–5 d at 30 °C (BTL Ltd., Łódź, Poland);
7. The presence of *Salmonella*, on SS agar (*Salmonella–Shigella* and XLD) after prior multiplication of samples in buffered peptone water and Rappaport–Vassiliadis broth (BTL Ltd., Łódź, Poland) for 24 h at 37 °C. Final identification was carried out using API tests (BioMerieux, Marcy l’Etoile, France) and polyvalent sera (Biomed Inc., Kraków, Poland).

Each sample was plated in triplicate. Following incubation, the colonies were counted with a Scan 300 automatic counter (Interscience, Saint Nom la Brétèche, France) and enumerated in accordance with ISO 4832 [30] and ISO 7218 [31]. Next, the numbers of each morphological type were determined and expressed as colony forming units per g of intestinal contents [CFU/g].

### 2.4. Statistical Analysis

The results obtained in each group were presented as arithmetic means (M) and standard deviations (SDs) as well as the standard error of the mean (SEM). The normality of the distribution was tested by the Shapiro–Wilk test. If the distribution was normal, one-way analysis of variance was performed, and the differences between groups were determined by the Tukey test. The analysis was performed using Statistica software version 13.1 (StatSoft Inc., Tulsa, OK, USA).

Values were considered to differ significantly at *p* ≤ 0.05. Values designated with the same superscript letters a, b between groups differ significantly (*p* ≤ 0.05). Correlations marked with an asterisk (*) are statistically significant (*p* ≤ 0.05).

## 3. Results

### 3.1. Chemical Composition of RSM before and after Fermentation

The effect of fermentation on the chemical composition of the RSM is presented in Table 2. The fermentation process caused a minor increase in the content of protein and ash and a decrease in the content of fat, fibre and dry matter. The increase in the content of protein and lactic acid can be explained by the bacteria accompanying fermentation and by protein synthesis from the biomass of microbes (Table 2).

### 3.2. Microbial Population in the GIT of Rabbits

Microbiological diagnostics of the bacterial microbiota of the GIT is a valuable parameter in assessment of the bacterial balance of the intestines. The microbial activity of the intestinal contents was determined as the total numbers of microbes of each group, including the number of aerobic and anaerobic bacteria, as well as their concentrations in each part of the GIT (Table 3). The number of mesophilic aerobic bacteria in the duodenum, caecum and colon was similar in all groups. A statistically significant increase in the number of mesophilic aerobic bacteria was observed only in the contents of the small intestine of group FR4 relative to the control (group C). The concentration of microscopic fungi was similar in all experimental groups and in the control group (Table 3).

Analysis of the numbers of coliforms did not reveal any significant effect of FRSM on their concentration in the duodenum or caecum (Table 4). In the small intestine, on the other hand, there was a gradual decrease in the number of coliforms that was significantly lower in the group of rabbits receiving a diet with 12% FRSM (FR12) than in the control (*p* < 0.05). In the colon, the increase in the number of coliforms in the control group was statistically significant in comparison to all experimental groups (FR4, FR8 and FR12). A similar relationship was noted for *Escherichia coli* bacteria (Table 4). A statistically significant increase in the number of *E. coli* bacteria was noted in the small intestine of the animals in group C compared to groups FR8 and FR12 (*p* < 0.05) and in the colon of animals in group C relative to the other experimental groups.

In the duodenum of the rabbits receiving a diet with FRSM (FR4, FR8 and FR12), there was a statistically significant increase in the number of lactic acid bacteria (LAB) relative to group C. In the small intestine and colon, a significant increase in LAB was noted in the groups receiving 8% and 12% FRSM (Table 4).

The number of anaerobic bacteria of the species *Clostridium perfringens* in the duodenum and caecum of the animals in the control group was statistically significantly higher than in the animals whose feed contained FRSM (Table 5). The diet with 12% FRSM (group FR12) caused a statistically significant (*p* < 0.05) decrease in the number of *Clostridium perfringens* bacteria in the contents of the small intestine and colon relative to the control group and other experimental groups (Table 5).

Pearson correlation analysis of the numbers of microorganisms of various groups in different sections of the GIT showed numerous significant effects of lactic acid bacteria on the other microbial populations. In the duodenum, a strong negative correlation was observed between the number of lactic acid bacteria and the number of bacteria of the species *Clostridium perfringens* (−0.748, FR12), and in the control group between the number of LAB and the population of mesophilic aerobic bacteria (−0.986, C). Correlation analysis showed a very strong relationship (0.999) between the concentration of lactic acid bacteria in the duodenum and the number of fungi in this section of the GIT in group C (Table 6). In the caecum of rabbits in group FR8, a strong positive correlation (0.797) was noted between the number of lactic acid bacteria and the total number of mesophilic aerobic bacteria. Statistically significant relationships between LAB in the caecum contents and the number of *Escherichia coli* bacteria were confirmed by a strong negative correlation (−0.975). Similar relationships were noted in the small intestine of groups FR8 (−0.998) and FR12 (0.606). The number of lactic bacteria was lowest in the colon of rabbits in group C and was statistically significant. A very strong positive correlation was noted between the numbers of LAB and *Clostridium perfringens*: 0.877 for group C and 0.925 for FR4 (Table 6).

### 3.3. Titres of IgA, Ig and IgM in Rabbit Plasma

The level of class G immunoglobulins in the plasma of rabbits from group FR12 was statistically lower than in the control group C. The level of IgG decreased as the level of FRSM in the diet increased (Table 7). The statistical analysis showed no differences in the titres of class A and M immunoglobulins between groups (Table 7). However, it is worth noting the lower IgA and IgM values in the plasma of the rabbits in group FR4 compared to the other groups (*p* > 0.05).

Correlations were also sought between the level of class G immunoglobulins in the plasma and the numbers of microorganisms in the GIT of rabbits depending on the amount of FRSM in their diet. The Pearson analysis showed numerous complex correlations (Table 8). The table shows that the increase in intake of FRSM was correlated with an increase in the correlations between the immunoglobulin level and the size of the microbial population in the GIT. The concentration of class G immunoglobulins in the control group was strongly negatively correlated with the number of anaerobic *C. perfringens* bacteria in the duodenum (Table 8). In this part of the intestine, the IgG level was also strongly correlated (0.980) with the number of *E. coli* (FR8). In group FR12, a strong positive correlation (0.897) was noted between the concentration of IgG and the number of fungi. These antibodies in the colon of group C were strongly correlated with the number of mesophilic aerobic bacteria (0.970), the total number of fungi (0.989) and the number of *E. coli* (0.988).

## 4. Discussion

An essential factor for maintaining a balance of the populations of the microbiota is a diet with an appropriate composition. Disturbances of their activity may result from the presence of anti-nutritional substances in feed components such as glucosinolates or tannins in RSM. During fermentation, anti-nutritional substances undergo reduction, leading to an increase in the level of lactic acid that exerts a beneficial effect by stimulating the immune system. FRSM, owing to its nutritional value and health-promoting properties, can beneficially affect production parameters, nutrient digestibility, the gut microbiota and metabolic processes [18].

During digestion in rabbits, changes take place in physicochemical parameters, such as pH, redox potential, and metabolite concentrations, that directly affect the balance of microbial communities in the GIT [32]. Michelland et al. [33] demonstrated that the bacterial community of the caecum of rabbits can change and adapt to varying qualities and quantities of nutrients in the diet and can achieve a balance quickly following intake of new feed. This was confirmed in the present study in which the inclusion of FRSM in the diet of rabbits was not shown to affect most key groups of microorganisms taking part in feed digestion.

The microbial species variation in the GIT of rabbits is affected by numerous factors, from intestinal peristalsis and mixing of the intestinal contents determined by physiological structure, to periodic gastrointestinal strictures (as in the duodenum), to the presence of a number of gastrointestinal hormones and peptides, such as cholecystokinin, somatostatin, vasoactive intestinal peptide and “substance P” [34]. Bowel transit time is highly variable and shorter than in other herbivores. Peristaltic contractions occur slowly, every 10–15 min, and do not change with the stages of the caecotrophic cycle. Food retention times are estimated at 10–20 min in the jejunum and 30–60 min in the ileum [35]. The type and number of microorganisms in the GIT depends on the section of the GIT. In its first sections, i.e., the stomach and superior part of the duodenum, due to their acidic pH, the presence of bile, shorter transit time and limited mucus production, the number of microbes is smaller. These sections of the GIT are dominated by lactobacilli and enterococci. From the superior part of the duodenum to the jejunum and ileum, the number of bacteria steadily increases, until it reaches a value from 10^11^ to 10^12^ CFU/g of faeces in the colon and caecum. The availability of oxygen and changes in pH in different sections of the GIT are conducive to the development of *Bacteroides* spp., which carry out fermentation processes leading to the generation of volatile fatty acids. These, in turn, regulate intestinal pH, serve as an energy source for intestinal epithelial cells and promote gastrointestinal motility [36].

A high level of high-quality fibre (NDSF) and protein in the diet of rabbits is important as well. Intake of rapidly fermenting fibre has a beneficial effect on the activity and concentration of volatile fatty acids in the caecum and improves the functioning of the intestinal mucosa and the structure of the microbiota. A diet with a well-balanced content of protein and essential amino acids, rich in arginine, reduces the population of *Clostridium* spp. and *Helicobacter* spp. bacteria in the intestines, thereby reducing mortality in the herd [37]. The diet proposed in the present study, containing FRSM (8–12%), can be a valuable source of these substances that benefit rabbit health and can play a role in preventing imbalances in the gut microbiota. The probiotic and prebiotic substances contained in it are believed to stimulate the growth of *Bifidobacterium* and *Lactobacillus* bacteria, which are beneficial for the host. Probiotics have been shown to have a positive effect on the gut microbiota of rabbits in research by Bónai et al. [38]. The authors observed a decrease in the number of anaerobic *Clostridium* spp. and *E. coli* bacteria following administration of inulin. These results are consistent with those obtained in the present study. Rabbits from the groups receiving a diet with an increased proportion of FRSM (8% or 12%) had lower concentrations of anaerobic bacteria and *E. coli* in the intestinal contents. Bacteria of the genera *Bacillus* and *Lactobacillus*, used in fermentation of rapeseed, may perform an important probiotic role in the GIT of rabbits. The biological effects of their activity depend on the strains of microorganisms and their ability to maintain metabolic activity in the gut environment. Studies by Bónai et al. [39] and Pascual et al. [40] indicate that the addition of a probiotic in the form of *Bacillus cereus* bacteria or *Saccharomyces cerevisiae* yeast to the diet of rabbits has a beneficial effect on their health. The authors observed a decrease in the number of *Clostridium* and *E. coli* bacteria, which is in agreement with the results of our experiment. Research by Kimse et al. [41], on the other hand, found that probiotic bacteria did not affect the structure or diversity of the bacterial population in the intestine of rabbits. In the present study as well, the probiotic microorganisms contained in the FRSM had no significant effect on the size of the major groups of microorganisms or their total number. Although the occurrence of *Lactobacillus* strains in the rabbit microbiota is low, supplementation with them increases the number of cellulolytic bacteria and increases the number of ureolytic bacteria [42]. In the present study, a significant increase in the number of lactic acid bacteria in the duodenum and colon was observed as the proportion of FRSM in the diet increased.

The gut microbiota is closely linked to the immune system (gut-associated lymphoid tissue—GALT) and in most mammals is primarily located in the small intestine and colon. Rabbits have special additional structures—the sacculus rotundus, located at the ileocaecal junction, and the vermiform appendix, at the end of the caecum. Together with the gut microbiota, lymphoid aggregates, such as Peyer’s patches and isolated cells dispersed in the lamina propria and the epithelium of the villi, play an important role in the acquisition of immunity and differentiation of antibodies [43]. FRSM, rich in bioactive peptides, can stimulate the activity of the intestinal immune system (GALT). By stimulating the microbiota of the GIT and reducing the proportion of potentially pathogenic microbes, such as *Clostridium* or *E. coli* bacteria, it can counteract excessive immunisation in the intestinal mucosa and prevent inflammation. This is confirmed by Stappenbeck et al. [44], who observed a decrease in GALT activity in mice that were not exposed to pathogens. The authors also showed weak immunisation of the immune system and a low level of antibodies in the blood serum. In rabbits, levels of antibodies are dependent on the gut microbiota, which influences the level and diversification of the repertoire of antibodies. A study by Severson et al. [45] showed that the GALT system, despite continual immunisation by the commensal bacteria of the digestive tract, is able to acquire tolerance to *Bacillus* antigens with no increase in the immune response. This is confirmed by the present study using RSM fermented with *Bacillus* strains. This suggests that tolerance to the antigens of these microbes can be acquired, and the strength and direction of the effect of FRSM was established based on correlation analysis. The formation of mechanisms of tolerance also depends on the presence of probiotic microorganisms and the substances they produce.

FRSM, by supplying valuable bioactive substances, appears to be a good component in the diet of rabbits, enhancing immune system development and helping to prevent disturbances of the gut microbiota. The results of the present study indicate that the use of new biotechnological research solutions can lead to changes in agricultural practices. The use of fermented feed products in the diet can diversify protein sources and stimulate the beneficial microbiota of the GIT of animals. These measures are particularly important because they can help to reduce the use of antibiotics for therapeutic purposes through nutritional prevention and optimal development of the immune system.

## Figures and Tables

**Table 1:** Ingredients (%) and chemical composition of feed for rabbit.

**Ingredient (%)**	**C**	**FR4**	**FR8**	**FR12**
Maize	11.00	10.22	9.32	8.42
Barley	24.95	24.95	24.95	24.95
Wheat bran	18.2	18.2	18.2	18.2
SBM, 46.5% CP	10.85	7.85	4.88	1.90
Monocalcium phosphate	0.915	0.831	0.748	0.665
Salt	0.443	0.424	0.404	0.385
Mineral-vitamin premix ^1^	1	1	1	1
Milk replacer ^2^	2	2	2	2
Dried alfalfa	30	30	30	30
Coccidiostat	0.05	0.05	0.05	0.05
Fodder chalk	0.641	0.520	0.498	0.477
FRSM	-	4	8	12
**Content of analysed nutrients and bioactive substances in 1 kg of rabbit diets (*n* = 3)**				
Metabolizable energy ^3^, MJ	10.47	10.42	10.35	10.32
Dry matter, g	885.7	885.5	885.3	885.2
Crude fibre, g	130.09	130.30	130.45	130.80
Crude protein, g	168.0	167.9	167.7	167.6
Ether extract, g	33.97	34.01	34.20	34.39
Crude ash, g	72.98	71.93	71.87	71.82
Lysine, g	7.64	7.32	7.00	6.68
Methionine + cysteine, g	5.20	5.31	5.42	5.53
Total calcium, g	8.39	8.35	8.36	8.00
Total phosphorus, g	7.16	7.11	7.09	7.09
Phytate phosphorus, g	4.83	4.49	4.39	4.28
Total sodium, g	2.35	2.37	2.36	2.35
Lactic acid, g	0.791	6.00	7.48	9.03
Glucosinolates, mmol	0.077	0.409	0.798	1.30
Phytase, U/L	310	500	680	750
Tannin, g	1.89	2.44	3.18	3.71

^1^ Mineral and vitamin (content in 1 kg): vitamin A, 100,000 IU; vitamin D3, 150 IU; vitamin E, 5000 mg; vitamin K3, 150 mg; vitamin B1, 150 mg; vitamin B2, 500 mg; vitamin B6, 300 mg; vitamin B12, 1.5 µg; biotin, 20 mg; calcium, 20 g; manganese, 5 g; zinc, 5 g; iron, 5 g; copper, 0.6 g; selenium, 8 mg. ^2^ Milk replacer (content in 1 kg): crude protein, 190 g; crude fat, 120 g; crude fibre, 7 g; L-metioninę 4.7 g; L-lysine, 12 g; vitamin A, 27,000 IU; vitamin D3, 6000 IU; vitamin E, 80 mg; vitamin K3, 1 mg; vitamin B1, 8 mg; vitamin B2, 6 mg; vitamin B6, 4 mg; vitamin B12, 200 µg; vitamin C, 50 mg; Biotin, 60 µg; DL-α-Tocopherol 73 mg; calcium, 10 g; sodium, 6 g; phosphorus, 6 g; magnesium 1 g; iron, 100 mg; iodine 1 mg; copper, 10 mg; manganese, 40 mg; zinc, 75 mg; selenium 0.2 mg. ^3^ Metabolizable energy was calculated according to the equation of Kirchgessner and Roth [22]. SBM—soybean meal; FRSM—fermented rapeseed meal. Groups: C—control; FR4—group receiving a diet with 4% FRSM; FR8—group receiving a diet with 8% FRSM; FR12—group receiving a diet with 12% FRSM.

**Table 2:** Mean content of nutrients in 1 kg of feed (g).

Parameter	RSM	FRSM
Dry matter, g	883.5	878.7
Crude fibre, g	143.75	141.55
Crude protein, g	337.50	361.45
Ether extract, g	24.05	16.3
Crude ash, g	70.7	78.15
Lysine, g	13.07	13.08
Methionine + cysteine, g	12.21	12.94
Total calcium, g	7.38	7.91
Total phosphorus, g	9.67	10.91
Phytate phosphorus, g	3.97	3.16
Total sodium, g	1.91	1.98
Lactic acid, g	6.27	38.83
Glucosinolates, mmol	21.98	11.40
Phytase, U/L	76.73	112.3
Tannin, g	10.02	7.13

RSM—rapeseed meal; FRSM—fermented rapeseed meal.

**Table 3:** Numbers of mesophilic aerobic bacteria and fungi in each section of the GIT [log CFU/g contents].

Section of GIT	C	FR4	FR8	FR12	SEM
	M ± SD	M ± SD	M ± SD	M ± SD	
**Number of mesophilic aerobic bacteria**					
Duodenum	4.2 ± 0.19	4.2 ± 0.10	4.3 ± 0.12	4.4 ± 0.30	0.084
Small intestine	4.9 ^b^ ± 0.78	6.5 ^a^ ± 0.41	5.2 ^ab^ ± 0.58	5.8 ^ab^ ± 0.81	0.216
Caecum	4.6 ± 0.24	4.5 ± 0.25	4.5 ± 0.29	4.9 ± 0.27	0.073
Colon	4.4 ± 0.51	4.4 ± 0.11	4.4 ± 0.12	4.9 ± 0.25	0.085
**Total number of fungi**					
Duodenum	1.5 ± 1.00	0.5 ± 1.00	0.5 ± 1.00	0.5 ± 1.00	0.250
Small intestine	1.5 ± 1.00	0.5 ± 1.00	0.5 ± 1.00	0.5 ± 1.00	0.250
Caecum	0.8 ± 1.50	1.3 ± 1.50	0.5 ± 1.00	1.3 ± 1.50	0.322
Colon	0.5 ± 1.00	0.5 ± 1.00	0.5 ± 1.00	0.5 ± 1.00	0.224

M—Arithmetic mean; SD—standard deviation; SEM—standard error of the mean. ^a,b^—Values in rows marked with different letters differ significantly at *p* ≤ 0.05. GIT—gastrointestinal tract. Groups: C—control; FR4—group receiving a diet with 4% FRSM; FR8—group receiving a diet with 8% FRSM; FR12—group receiving a diet with 12% FRSM.

**Table 4:** Numbers of coliforms, *E. coli* and lactic acid bacteria in each section of the GIT [log CFU/g contents].

Section of GIT	C	FR4	FR8	FR12	SEM
	M ± SD	M ± SD	M ± SD	M ± SD	
Number of coliforms					
Duodenum	4.2 ± 0.40	3.3 ± 0.47	3.6 ± 0.28	2.1 ± 2.42	0.345
Small intestine	4.2 ^a^ ± 0.44	3.04 ^ab^ ± 2.05	1.25 ^ab^ ± 1.50	1.15 ^b^ ± 1.35	0.464
Caecum	2.5 ± 1.74	1.90 ± 2.19	0.87 ± 1.73	1.54 ± 1.78	0.447
Colon	3.9 ^a^ ± 0.180	1.2 ^b^ ± 1.50	0.5 ^b^ ±1.00	0.5 ^b^ ± 1.00	0.428
Number of *E. coli*					
Duodenum	4.2 ± 0.19	1.9 ± 2.23	2.0 ± 2.28	1.9 ± 2.17	0.503
Small intestine	4.0 ^a^ ± 0.11	1.2 ^ab^ ± 2.28	0.5 ^b^ ± 1.00	0.5 ^b^ ± 1.00	0.477
Caecum	3.1 ± 0.24	1.5 ± 1.69	1.1 ± 1.31	1.2 ± 1.38	0.354
Colon	3.6 ^a^ ± 0.04	0.7 ^b^ ± 1.30	0.6 ^b^ ± 1.15	0.6 ^b^ ± 1.15	0.410
Number of lactic acid bacteria					
Duodenum	1.9 ^b^ ± 1.28	3.9 ^a^ ± 0.32	3.9 ^a^ ± 0.25	4.3 ^a^ ± 0.27	0.282
Small intestine	0 ± 0.00c	2.1 ^b^ ± 1.44	3.9 ± 0.41a	4.8 ^a^ ± 0.39	0,506
Caecum	3.6 ± 0.49	4.1 ± 0.04	4.3 ± 0.05	4.4 ± 0.69	0.122
Colon	2.3 ^b^ ± 1.50	3.7 ^ab^ ± 0.09	4.0 ^a^ ± 0.08	4.5 ^a^ ± 0.23	0.278

M—Arithmetic mean; SD—standard deviation; SEM—standard error of the mean. ^a,b^—Values in rows marked with different letters differ significantly at *p* ≤ 0.05. GIT—gastrointestinal tract. Groups: C—control; FR4—group receiving a diet with 4% FRSM; FR8—group receiving a diet with 8% FRSM; FR12—group receiving a diet with 12% FRSM.

**Table 5:** Number of *Clostridium perfringens* bacteria in each section of the GIT [log CFU/g contents].

Section of GIT	C	FR4	FR8	FR12	SEM
	M ± SD	M ± SD	M ± SD	M ± SD	
Number of mesophilic aerobic bacteria					
Duodenum	5.8 ^a^ ± 0.37	4.5 ^b^ ± 0.15	3.2 ^b^ ± 2.13	4.8 ^b^ ± 0.28	0.343
Small intestine	4.9 ^a^ ± 0.26	4.6 ^a^ ± 0.28	4.6 ^a^ ± 0.24	3.3 ^b^ ± 0.38	0.172
Caecum	5.8 ^a^ ± 0.40	5.0 ^b^ ± 0.08	4.3 ^c^ ± 0.23	4.9 ^bc^ ± 0.33	0.150
Colon	4.6 ^a^ ± 0.04	4.0 ^a^ ± 0.09	3.0 ^a^ ± 0.25	2.5 ^b^ ± 1.7	0.282

M—Arithmetic mean; SD—standard deviation; SEM—standard error of the mean. ^a,b^—Values in rows marked with different letters differ significantly at *p* ≤ 0.05. GIT—gastrointestinal tract. Groups: C—control; FR4—group receiving a diet with 4% FRSM; FR8—group receiving a diet with 8% FRSM; FR12—group receiving a diet with 12% FRSM.

**Table 6:** Correlations among numbers of microorganisms of different groups in sections of the GIT.

Section of the GIT				
Parameter/Group	C	FR4	FR8	FR12
**Duodenum**				
LAB × *Clostridium perfringens*	−0.190	−0.564	0.218	−0.748 *
LAB × total bacterial count	−0.986 *	0.678	−0.739 *	−0.881
LAB × yeast	0.999 *	0.758	0.756	0.406
**Small Intestine**				
LAB × yeast	0.00	−0.988 *	0.684	0.082
LAB × *Escherichia coli*	0.00	−0.339	0.084	**−0.606** *
**Caecum**				
LAB × total bacterial count	−0.920	−0.402	0.797 *	−0.433
LAB × *E. coli*	−0.862	0.352	−0.975 *	0.343
**Colon**				
LAB × *Clostridium perfringens*	0.877 *	0.925 *	0.078	0.727
LAB × total bacterial count	0.267	−0.627 *	−0.879 *	−0.411

* Statistically significant correlations in bold with asterisk. GIT—gastrointestinal tract. Groups: C—control; FR4—group receiving a diet with 4% FRSM; FR8—group receiving a diet with 8% FRSM; FR12—group receiving a diet with 12% FRSM.

**Table 7:** Titre of immunoglobulins in rabbit plasma (ng/mL).

Section of GIT	C	FR4	FR8	FR12	SEM
	M ± SD	M ± SD	M ± SD	M ± SD	
IgA	0.08 ± 0.06	0.06 ± 0.02	0.09 ± 0.01	0.08 ± 0.01	0.007
IgM	0.76 ± 0.06	0.68 ± 0.08	0.62 ± 0.15	0.74 ± 0.04	0.024
IgG	2.33 ^a^± 0.67	1.84 ^ab^ ± 0.85	1.49 ^ab^ ± 0.49	1.15 ^b^ ± 0.35	0.178

M—Arithmetic mean; SD—standard deviation; SEM—standard error of the mean. ^a,b^—Values in rows marked with different letters differ significantly at *p* ≤ 0.05. GIT—gastrointestinal tract. Groups: C—control; FR4—group receiving a diet with 4% FRSM; FR8—group receiving a diet with 8% FRSM; FR12—group receiving a diet with 12% FRSM.

**Table 8:** Correlation between IgG and microorganisms in the intestine.

Section of the GIT				
Parameter/Group	C	FR4	FR8	FR12
**Duodenum**				
Number of anaerobic *C. perfringens*	−0.864 *	0.487	−0.672	−0.126
Number of *E. coli*	−0.409	0.103	0.980*	−0.536
**Small Intestine**				
Number of anaerobic *C. perfringens*	−0.962 *	−0.625	0.445	0.375
Total number of bacteria	0.970 *	−0.041	0.616	−0.102
**Caecum**				
Total number of fungi	−0.372	0.349	0.443	0.897 *
**Colon**				
Total number of mesophilic aerobic bacteria	0.991 *	−0.586	−0.361	0.493
Total number of fungi	0.989 *	−0.538	0.443	−0.325
Number of *E. coli*	0.988 *	−0.538	0.443	−0.098

* Statistically significant correlations in bold with asterisk. GIT—gastrointestinal tract. Groups: C—control; FR4—group receiving a diet with 4% FRSM; FR8—group receiving a diet with 8% FRSM; FR12—group receiving a diet with 12% FRSM.

## Data Availability

All relevant data are within the paper.
